# Genome-Wide Characterization of QYYZ-Like PRRSV During 2018–2021

**DOI:** 10.3389/fvets.2022.945381

**Published:** 2022-06-30

**Authors:** Hu Xu, Lirun Xiang, Yan-Dong Tang, Chao Li, Jing Zhao, Bangjun Gong, Qi Sun, Chaoliang Leng, Jinmei Peng, Qian Wang, Guohui Zhou, Tongqing An, Xuehui Cai, Zhi-Jun Tian, Hongliang Zhang, Mingxin Song

**Affiliations:** ^1^College of Veterinary Medicine, Northeast Agricultural University, Harbin, China; ^2^State Key Laboratory of Veterinary Biotechnology, Harbin Veterinary Research Institute, Chinese Academy of Agricultural Sciences, Harbin, China; ^3^Henan Key Laboratory of Insect Biology in Funiu Mountain, Henan Provincial Engineering Laboratory of Insect Bioreactors, China-UK-NYNU-RRes Joint Laboratory of Insect Biology, Nanyang Normal University, Nanyang, China

**Keywords:** QYYZ-like PRRSV, whole-genome analysis, complex patterns of recombination, recombination hotspots, epidemiological characteristics

## Abstract

In the last decade, the emergence of QYYZ-like porcine reproductive and respiratory syndrome virus (PRRSV) has attracted increasing attention due to the high incidence of PRRSV mutation and recombination. However, the endemic status and genomic characteristics of the QYYZ-like strains are unclear. From 2018 to October 2021, 24 QYYZ-like PRRSV isolates were obtained from 787 PRRSV-positive clinical samples. Only one QYYZ-like positive sample was from a northern province, and the rest were from central and southern provinces. We selected 9 samples for whole-genome sequencing, revealing genome lengths of 15,008–15,316 nt. We retrieved all the available whole-genome sequences of QYYZ-like PRRSVs isolated in China from 2010 to 2021 (*n* = 28) from GenBank and analyzed them together with the new whole-genome sequences (*n* = 9). Phylogenetic tree analysis based on the ORF5 gene showed that all QYYZ-like PRRSV strains belonged to sublineage 3.5 but were clustered into three lineages (sublineage 1.8, sublineage 3.5, and sublineage 8.7) based on whole-genome sequences. Genomic sequence alignment showed that QYYZ-like strains, have characteristic amino acids insertions or deletions in the Nsp2 region (same as NADC30, JXA1 and QYYZ) and that thirteen strains also had additional amino acid deletions, mostly between 468 and 518 aa. Moreover, QYYZ-like strains (sublineage 3.5) have seven identical characteristic amino acid mutations in ORF5. Recombination analysis revealed that almost all QYYZ-like complete genome sequences (36/37) were products of recombination and mainly provided structural protein fragments (GP2-N) for the recombinant strains. Overall, QYYZ-like strains were mainly prevalent in central and southern China from 2018 to 2021, and these strains provided recombinant fragments in the PRRSV epidemic in China.

## Introduction

Porcine respiratory and reproductive syndrome (PRRS) is a major disease in the pig industry that causes huge economic losses to the swine industry worldwide (1). The causative agent, porcine reproductive and respiratory syndrome virus (PRRSV), is an enveloped, positive-sense, single-stranded RNA virus belonging to the genus *Betaarterivirus* and family *Arteriviridae* of order *Nidovirales* (2). Due to its high degree of genetic diversity, PRRSV has been further divided into two species, PRRSV-1 (formerly known as European genotype 1) and PRRSV-2 (formerly known as North American genotype 2) (3). Since PRRSV was first discovered in China in 1996, the PRRSV-2 strain has been the main circulating strain in China (4).

Phylogenetic analyses of ORF5 of PRRSV-2 strains showed that PRRSV-2 could be divided into nine distinct lineages, with each lineage containing several sublineages (5, 6). Four different PRRSV-2 lineages have become widespread in China, including lineage 8 (JXA1-like/CH-1a-like), lineage 5 (VR-2332-like), lineage 1 (NADC30-like/NADC34-like), and lineage 3 (QYYZ-like) (7). The first PRRSV-2 strain CH-1a of China was isolated in 1996 in lineage 8 (8). HP-PRRSV (JXA1-like) was recognized in 2006 and originated from CH-1a-like strains (9). In 2013, some NADC30-like strains were isolated in China, and they have gradually become the dominant strains in recent years (10). NADC34-like strains were first reported in China in 2017 and became one of the major epidemic strains in 2020 (7, 11). Lineage 3 strains were initially reported in Taiwan and have emerged in Hong Kong (12). The FJ-1 strain was the first lineage 3 PRRSV detected in mainland China in 2005. The representative isolate of lineage 3 was QYYZ, which was identified in 2010 in mainland China and gradually became prevalent in southern China (12, 13). More importantly, lineage 3 viruses with greater virulence have been reported in southern China, and these viruses, recombining with lineage 1 and 8 PRRSV, pose a great threat to the Chinese pig industry (14–17).

Several studies have summarized the origin, classification, epidemic history and population dynamics of QYYZ-like strains based on the ORF5 gene (18, 19). However, the prevalence and genomic characteristics of these strains in recent years remain unclear. In this study, we carried out molecular epidemiological investigations for QYYZ-like PRRSV surveillance from 2018 to 2021. Meanwhile, the genome-wide characteristics of QYYZ-like strains and the role of these strains in the PRRSV epidemic were explored by comparing the latest strains and all reported genome-wide sequences.

## Materials and Methods

### Sample Collection and Genome Sequencing

From 2018 to 2021, we collected 1,803 clinical samples (including lung, lymph node and serum samples) of suspected PRRSV infection from different pig farms in 16 provinces in China (Heilongjiang, Jilin, Liaoning, Shandong, Henan, Guangdong, Guangxi, Zhejiang, Hebei, Hubei, Xinjiang, Inner Mongolia, Tianjin, Sichuan, Jiangxi and Jiangsu). Tissue sample processing, RNA extraction, cDNA preparation, RT–PCR and genome sequencing were performed as described in previous reports (8, 11). The primers used to detect PRRSV and amplify entire gene sequences were reported previously (20).

### Sequence Analysis and Phylogenetic Analysis

Sequence analysis was performed with DNASTAR (version 7.1) software. Phylogenetic trees and molecular evolutionary analyses were conducted by MEGA 7 software using the neighbor-joining method with 1,000 bootstrap replications (21). The generated phylogenetic tree was annotated using the online software ITOL (https://itol.embl.de/) (22).

### Recombination Analysis

To determine whether recombination screening occurred in the generation of QYYZ-like PRRSV strains, recombination events were considered only when supported by at least four of seven recombination detection algorithms (RDP, GENECONV, BootScan, MaxChi, Chimera, SiScan and 3Seq) in the Recombination Detection Program version 4.8 (RDP v.4.8). Finally, the pictures of recombination events were drawn by SimPlot v3.5.1 within a 500-bp window sliding along the genome alignment (20-bp step size).

## Results and Discussion

**Figure 1:**
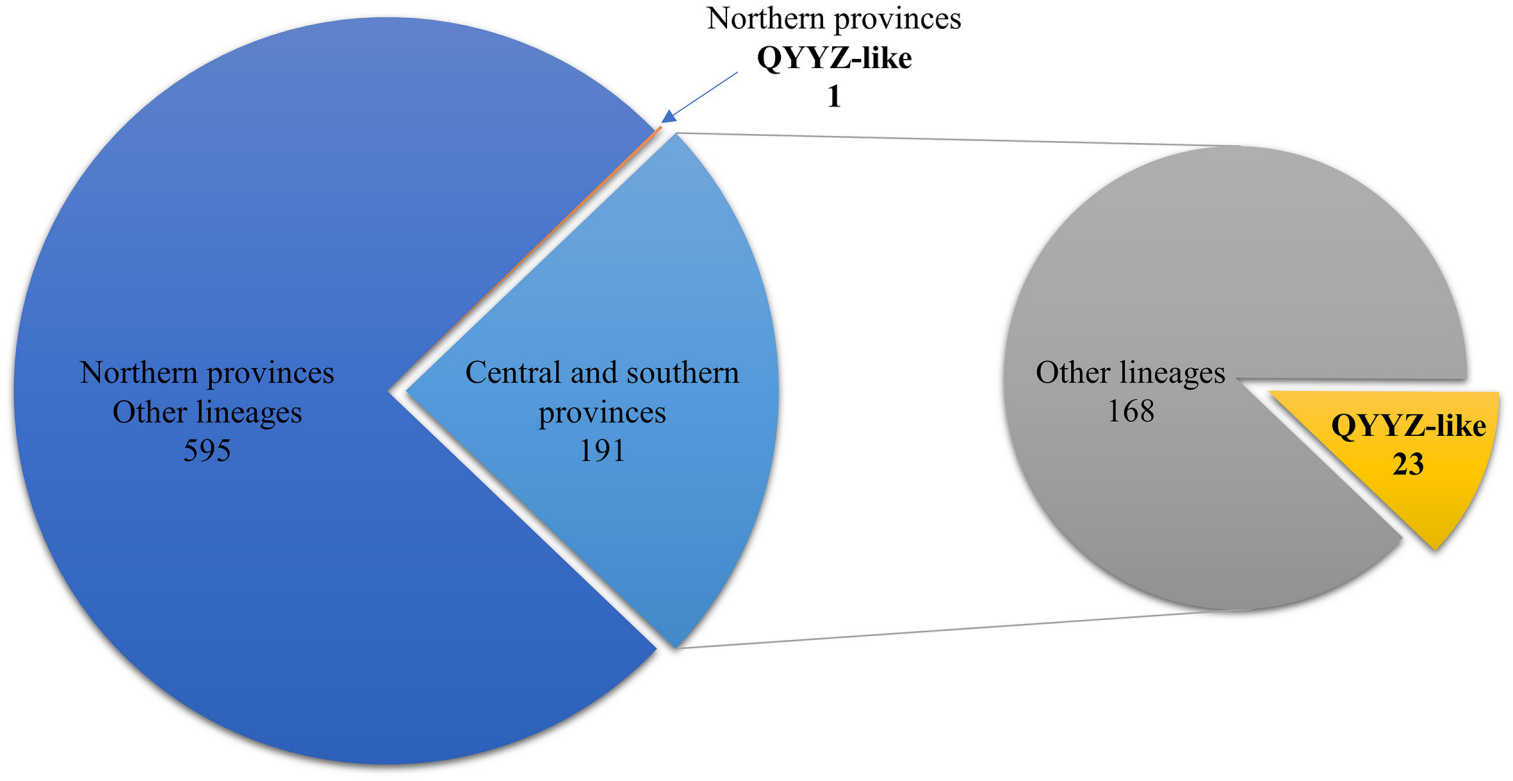
Number of QYYZ-like strains detected in different regions from 2018 to 2021. Among the 596 PRRSV-positive samples from northern provinces, 1 was a QYYZ-like strain. The remaining 191 PRRSV-positive samples were from central and southern provinces, of which 23 were QYYZ-like strains.

From 2018 to 2021, 1,803 clinical samples were collected from 16 provinces of China; 787 (43.64%) tested positive for PRRSV according to RT–PCR. Of the 787 positive samples, 191 were from central or southern provinces (Henan, Guangdong, Guangxi, Zhejiang, Hubei, Sichuan, Jiangsu, and Jiangxi), and the remaining 596 were from northern provinces (Heilongjiang, Jilin, Liaoning, Shandong, Hebei, Xinjiang, Inner Mongolia, and Tianjin) (Figure 1). Through ORF5 phylogenetic analysis, 24 samples were confirmed to have QYYZ-like PRRSV. The results showed that QYYZ-like PRRSV did not cause a pandemic in China but persisted during 2018–2021. Interestingly, almost all samples with QYYZ-like PRRSV were from central and southern provinces, including Henan (4), Guangdong (9), Zhejiang (1) and Guangxi (9), and only one QYYZ-like strain came from Heilongjiang Province (Table 1). The QYYZ-like strains accounted for approximately 12% (23/191) of the cases in the central and southern provinces (Figure 1). Therefore, QYYZ-like PRRSV was mainly prevalent in central and southern China. To further study the complete genome characteristics of QYYZ-like PRRSV in China, we selected 9 strains (HNLCL15-1903, GXXNF10-1803, GXXNF53-1805, GDXNF60-1805, GXXNF74-1806, GXXNF78-1806, GDXNF229-1811, HNTZJ1714-2011, GXTZJ2325-2112) from 24 newly identified QYYZ-like isolates based on large homology differences and different branches of an ORF5 phylogenetic tree for whole-genome sequencing. The genomes of these isolates were 15,008–15,316 nt in length, excluding 3′ poly (A) tails. The significant difference in gene length between the newly QYYZ-like PRRSV strains may be due to the different deletion or insertion patterns in the Nsp2 region.

**Table 1:** Information on 24 strains of the newly identified QYYZ-like PRRSV.

**No**.	**Isolates**	**Accession no**.	**Time**	**Isolation source**	**Province**	**Gene region**
1	GDXNF8-1802	ON462023	2018.02	Lung/lymph nodes	Guangdong	ORF5
2	GXXNF10-1803	ON462046	2018.03	Serum	Guangxi	Whole genome
3	GDXNF17-1804	ON462024	2018.04	Lung/lymph nodes	Guangdong	ORF5
4	GXXNF53-1805	ON462047	2018.05	Lung	Guangxi	Whole genome
5	GDXNF59-1805	ON462025	2018.05	Lung	Guangdong	ORF5
6	GDXNF60-1805	ON462048	2018.05	Lung	Guangdong	Whole genome
7	GXXNF74-1806	ON462049	2018.06	Lung	Guangxi	Whole genome
8	GXXNF75-1806	ON462026	2018.06	Lung	Guangxi	ORF5
9	GXXNF76-1806	ON462027	2018.06	Lung	Guangxi	ORF5
10	GXXNF77-1806	ON462028	2018.06	Lung/lymph nodes	Guangxi	ORF5
11	GXXNF78-1806	ON462050	2018.06	Lung/lymph nodes	Guangxi	Whole genome
12	GXXNF79-1806	ON462029	2018.06	Lung	Guangxi	ORF5
13	GDXNF134-1809	ON462030	2018.09	Lung	Guangdong	ORF5
14	ZJWK211-1809	ON462022	2018.09	Serum	Zhejiang	ORF5
15	GDXNF229-1811	ON462051	2018.11	Lung/lymph nodes	Guangdong	Whole genome
16	HNLCL15-1903	ON462043	2019.03	Serum/lung	Henan	Whole genome
17	HNLCL19-1904	ON462031	2019.04	Serum/lung	Henan	ORF5
18	HNLCL25-1905	ON462032	2019.05	Serum/lung	Henan	ORF5
19	HNLCL43-1906	ON462033	2019.06	Serum/lung	Henan	ORF5
20	GDHSW97-2001	ON462034	2020.01	Lung	Guangdong	ORF5
21	GDHSW100-2001	ON462035	2020.01	Lung	Guangdong	ORF5
22	HLJTZJ1224-2011	ON462036	2020.11	Lung	Heilongjiang	ORF5
23	HNTZJ1714-2011	ON462044	2020.11	Serum	Henan	Whole genome
24	GXTZJ2325-2112	ON462045	2021.12	Lung	Guangxi	Whole genome

To evaluate the genomic characteristics of the newly identified QYYZ-like PRRSV strains, the genomes of the novel PRRSV isolates were compared with those of different lineage viruses, including JXA1 (lineage 8), NADC30 (lineage 1) and QYYZ (lineage 3) (Table 2). Genome alignment revealed that the ORF5 gene of 24 QYYZ-like PRRSV strains shared 91.2–96.0% nucleotide homology with that of QYYZ, which was higher than the homology shared with that of JXA1 (81.9–85.7%) and NADC30 (82.2–85.2%). The homology among the 24 QYYZ-like strains was 86.4–100% (Table 2), and most of the QYYZ-like PRRSV ORF5 genes had low homology. The complete genome results also showed that the 9 newly identified QYYZ-like PRRSV strains shared 84.6–96.1% identity with JXA1, 82.3–90.8% identity with NADC30, and 83.6–93.8% identity with QYYZ (Table 2). Among them, the isolates GXXNF53-1805, GDXNF60-1805, GXXNF78-1806 and HNTZJ1714-2011 showed the highest identity (93.2, 95.4, 96.1, and 89.0%) with JXA1; the isolates GXXNF10-1803, GXXNF74-1806, GDXNF229-1811 and GXTZJ2325-2112 displayed the highest identity (87.8, 93.8, 91.0, and 86.3%) with QYYZ; and HNLCL15-1903 shared the highest identity with NADC30, but the homology was only 90.8%, suggesting that the newly identified QYYZ-like PRRSVs may have undergone large variation or recombination.

**Table 2:** Nucleotide and amino acid sequence similarity between the 24 new QYYZ-like PRRSVs and the reference strain.

**Amino acids/nucleotides**	**JXA1**	**NADC30**	**QYYZ**	**QYYZ-like (newly)**
5'UTR	–/96.8–98.9	–/91.0–93.1	–/94.1–95.2	–/94.1–99.5
Nsp1α	94.4–99.4/93.0–98.1	93.9–96.7/85.6–89.4	95.6–97.2/89.6–92.2	92.8–98.9/88.7–96.9
Nsp1β	87.6–95.5/89.3–97.0	73.7–75.8/75.7–80.2	79.7–82.7/81.5–84.3	84.7–95.0/88.3–96.2
Nsp2	70.9–96.3/76.0–97.3	68.6–88.1/73.6–90.4	68.0–87.3/74.7–91.4	67.3–92.9/73.4–94.3
Nsp3	86.1–99.1/81.3–98.1	87.0–96.5/80.6–93.6	87.8–98.3/80.9–94.3	86.1–99.1/80.1–96.1
Nsp4	90.7–100/81.9–98.5	89.2–93.1/81.4–93.8	91.7–97.5/83.5–95.4	90.2–99.0/81.0–97.5
Nsp5	87.6–95.9/80.2–97.1	86.5–96.5/80.2–94.3	87.6–97.1/80.6–95.3	86.5–95.3/78.8–94.1
Nsp6	93.8–100/85.4–100	93.8–100/83.3–97.9	93.8–100/83.3–97.9	93.8–100/81.2–100
Nsp7α	87.2–99.3/81.7–98.2	91.3–94.0/81.0–93.7	86.6–98.0/80.3–94.0	85.9–98.0/79.9–86.2
Nsp7β	71.8–100/77.9–99.1	70.9–90.9/77.6–92.7	75.5–94.5/78.5–94.5	69.1–100/75.8–98.2
Nsp8	93.3–100/88.1–98.5	91.1–95.6/83.0–94.8	93.3–100/88.1–94.1	88.9–100/83.7–97.8
Nsp9	96.6–99.5/85.4–98.9	96.0–98.3/85.3–95.0	95.0–98.1/84.4–92.5	94.9–99.1/84.3–97.5
Nsp10	93.7–99.5/84.5–98.6	94.6–97.7/84.8–95.1	93.0–99.8/84.6–98.1	92.7–99.3/84.1–97.2
Nsp11	92.8–98.2/85.9–95.7	91.9–96.3/84.0–91.2	93.7–97.8/86.1–96.7	91.5–96.9/83.3–96.9
Nsp12	96.1–100/86.4–98.9	92.9–96.1/85.3–90.1	93.5–99.4/85.7–97.4	92.9–99.4/84.0–98.1
ORF2a	86.0–90.3/87.8–92.0	87.2–88.3/84.2–87.4	92.6–96.5/91.7–97.0	88.7–94.6/89.0–94.7
ORF2b	86.5–91.9/91.0–94.6	83.8–91.9/88.7–91.4	91.9–98.6/94.1–96.4	87.8–94.6/90.5–94.6
ORF3	83.5–87.5/84.4–89.5	78.0–95.9/82.6–86.5	85.1–95.3/86.7–95.3	83.1–93.3/86.4–93.3
ORF4	85.5–96.1/84.7–94.4	88.3–91.6/86.0–88.5	87.2–96.1/86.2–95.2	87.2–94.4/84.5–92.7
ORF5	82.1–85.1/83.4–85.4	84.1–87.6/82.8–86.7	92.0–96.5/91.2–96.0	88.6–100/86.4–100
ORF5a	72.5–78.8/81.0–85.9	73.1–80.8/82.4–86.5	84.6–98.1/89.7–98.1	80.4–98.1/85.0–96.2
ORF6	94.3–96.6/89.0–91.2	92.0–95.4/88.2–90.9	96.0–98.9/92.8–97.5	95.4–98.9/90.3–96.4
ORF7	87.9–90.3/85.2–89.2	88.7–99.2/84.7–95.2	90.3–99.2/90.9–96.5	87.1–97.6/84.1–95.2
3'UTR	81.2–88.9	83.6–93.0	90.1–96.0	83.8–94.5
Whole genome	84.6–96.1	82.3–90.8	83.6–93.8	87.3–94.7

*The ORF5 homology results were for 24 newly identified PRRSV strains, and the other regional homology results were for 9 whole-genome sequences*.

**Figure 2:**
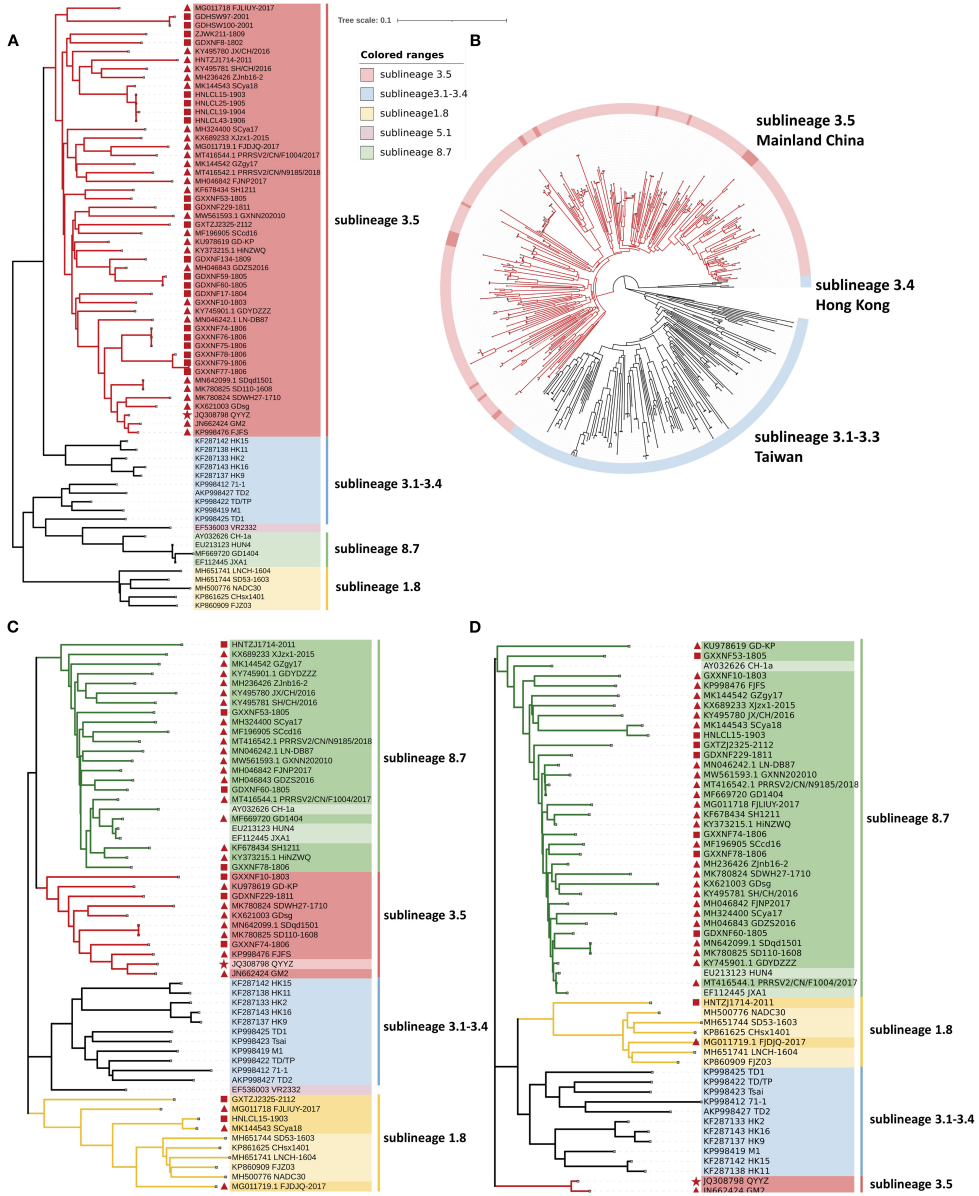
Phylogenetic analysis of QYYZ-like PRRSVs. **(A)** Phylogenetic tree constructed based on the ORF5 gene of novel QYYZ-like PRRSV isolates and reference PRRSV strains from each lineage. **(B)** Phylogenetic tree reconstructed based on the global data set (*n* = 470) of the lineage 3 PRRSV ORF5 genome. **(C)** Phylogenetic tree constructed based on full-length genomes of 37 QYYZ-like PRRSV isolates and reference PRRSV strains from each lineage. **(D)** Phylogenetic tree constructed based on the Nsp1 gene of 37 QYYZ-like PRRSV isolates and reference PRRSV strains from each lineage. The QYYZ-like prototype strain QYYZ is labeled with a red star (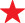). Newly obtained sequences in this study are labeled with red squares (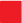). The QYYZ-like strains with complete genome sequences obtained from GenBank are labeled with red triangles (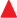).

To establish genetic relationships between Chinese QYYZ-like PRRSV strains and other PRRSV isolates, we constructed phylogenetic trees based on both the ORF5 gene and complete genomic sequence. Phylogenetic analysis based on ORF5 gene sequences demonstrated that all 24 new QYYZ-like PRRSV strains could be classified into sublineage 3.5 (Figure 2A). To analyze the genetic diversity of the novel QYYZ-like PRRSV in as much detail as possible, we expanded a data set (*n* = 470) including all ORF5 sequences of lineage 3 collected from GenBank and submitted before December 2021. As shown in Figure 2B, all 24 newly identified QYYZ-like strains in China (deep red labeled) clustered deeply within sublineage 3.5 from mainland China and did not form a separate clade. They are distantly related to sublineages 3.1–3.3 circulating in Taiwan and sublineage 3.4 circulating in Hong Kong (Figure 2B). To understand the genome-wide characteristics of QYYZ-like strains in China, we collected the whole-genome sequences of all QYYZ-like strains (ORF5 classified into sublineage 3.5) from GenBank (*n* = 28) (Table 3) and submitted before December 2021 and constructed phylogenetic trees together with the new strains (*n* = 9). Since the homology of QY2010 (Accession: JQ743666.1) and QYYZ (Accession: JQ308798.1) was up to 100% and the collection time of the two strains was very close, we regarded them as one strain and expressed them as QYYZ (Table 3). As shown in Figure 2C, 37 isolates were clustered into several branches with viruses belonging to different lineages. A total of 21 strains (GXXNF53-1805, GDXNF60-1805, GXXNF78-1806, HNTZJ1714-2011, etc.) were closely related to JXA1 and Hun4 (HP-PRRSV sublineage 8.7), while ten strains (GXXNF10-1803, GXXNF74-1806, GDXNF229-1811, etc.) were clustered into a separate branch close to QYYZ (sublineage 3.5). Additionally, HNLCL15-1903, GXTZJ2325-2112, SCya18 and FJLIUY-2017 were closer to NADC30 (sublineage 1.8). There was a large difference between the ORF5 gene-based and whole genome-based phylogenetic analyses. The epidemiology of PRRSV has been investigated largely by sequencing the ORF5 gene and classifying virus lineages based on ORF5 phylogenetic analysis (5, 6, 33). With the increasing number of recombinant strains, it is necessary to sequence the whole genome of key strains in order to classify them.

**Table 3:** Information on the whole genomes of all reported QYYZ-like PRRSV strains in China.

**Taxa**	**Isolation date**	**Recombination with**	**QYYZ-like regions**	**Accession no**.	**References**
GXXNF10-1803	2018.03	JXA1	Nsp2-Nsp8, ORF2-7	ON462046	This study
GXXNF53-1805	2018.05	JXA1	ORF2-ORF7	ON462047	This study
GDXNF60-1805	2018.05	JXA1	ORF2-ORF7	ON462048	This study
GXXNF74-1806	2018.06	JXA1	Nsp2-Nsp7, NSP9-ORF7	ON462049	This study
GXXNF78-1806	2018.06	JXA1	ORF2-ORF3, ORF5-ORF7	ON462050	This study
GDXNF229-1811	2018.11	JXA1	Nsp2-Nsp5, Nsp9-ORF7	ON462051	This study
HNLCL15-1903	2019.03	SH/CH/2016+NADC30	Nsp10-ORF6	ON462043	This study
HNTZJ1714-2011	2020.11	JXA1+NADC30	ORF2-ORF7	ON462044	This study
GXTZJ2325-2112	2021.12	JXA1+NADC30	Nsp4-ORF7	ON462045	This study
QYYZ	2010	-	-	JQ308798	(12)
QY2010	2010	-	-	JQ743666	(23)
GM2	2011	VR2332	Nsp1-Nsp8, Nsp11-ORF7	JN662424	(12)
SH1211	2012	JXA1	Nsp12-ORF2, ORF5-ORF6	KF678434	(24)
FJFS	2012	JXA1	Nsp2-ORF7	KP998476	(19)
GD1404	2014	JXA1	ORF4-ORF7	KT961415	(25)
HiNZWQ	2014	JXA1	ORF3-ORF6	KY373215	Not found
XJzx1-2015	2015	CH-1a	ORF5-ORF7	KR080330	(26)
GDsg	2015	JXA1	Nsp2-Nsp9, NSP10-ORF7	KX621003	(27)
SDqd1501	2015	JXA1	Nsp2-Nsp8, Nsp11-ORF7	MN642099	(28)
GD-KP	2015	JXA1+VR2332	Nsp2-Nsp3, Nsp5-NSP7, NSP11-ORF7	KU978619	(19)
SCcd16	2016	NADC30+JXA1	ORF5-ORF7	MF196905	(14)
GDZS2016	2016	JXA1	ORF2-ORF6	MH046843	(13)
ZJnb16-2	2016	JXA1	ORF2-ORF7	MH174986	(15)
SH/CH/2016	2016	JXA1	NSP12-ORF7	KY495781	(29)
JX/CH/2016	2016	JXA1	NSP12-ORF7	KY495780	(30)
SD110-1608	2016	JXA1	Nsp2-Nsp9, Nsp11-ORF7	MK780825	(19)
GDYDZZZ	2016	JXA1	NSP12-ORF7	KY745901	Not found
GZgy17	2017	JXA1	ORF2-ORF6	MK144542	(29)
FJNP2017	2017	JXA1	ORF2-ORF7	MH046842	(13)
SCya17	2017	JXA1+NADC30	ORF3-ORF5	MH324400	(14)
FJLIUY-2017	2017	NADC30+BJ4+JXA1	ORF5-ORF7	MG011718	(16)
SDWH27-1710	2017	JXA1	Nsp2-ORF7	MK780824	(19)
FJDJQ-2017	2017	NADC30	ORF2-ORF7	MG011719	Not found
LN-DB87	2017	JXA1	ORF2-ORF7	MN046242	(31)
PRRSV2/CN/F1004/2017	2017	JXA1	ORF4-ORF7	MT416544	(32)
SCya18	2018	SH/CH/2016 +NADC30	NSP11-ORF6	MK144543	(29)
PRRSV2/CN/N9185/2018	2018	NADC30+JXA1	ORF4-ORF7	MT416542	(32)
GXNN202010	2020	JXA1	ORF2-ORF7	MW561593	Not found

*Since the homology of QY2010 and QYYZ was up to 100% and the collection time of the two strains was very close, we regarded them as one strain and expressed them as QYYZ in the paper*.

**Figure 3:**
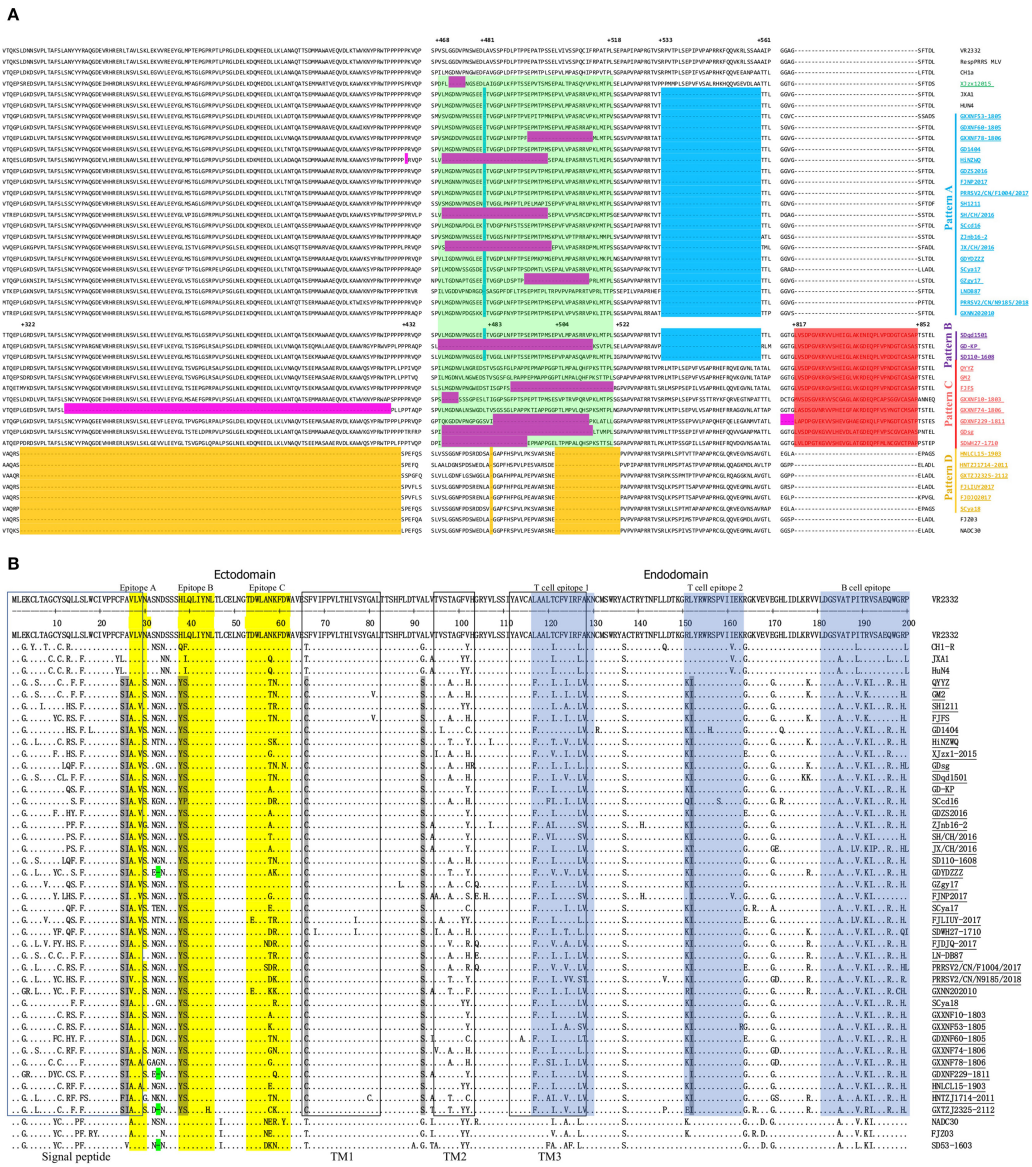
Alignment of the deduced amino acid sequences among QYYZ-like PRRSVs. **(A)** The positions marked in the figure represent positions of the Nsp2 amino acid sequence and refer to the position of VR-2332. Red indicates the QYYZ strain 36-aa characteristic continuous insertion; yellow indicates the NADC30-like PRRSV 131-aa characteristic discontinuous deletion; blue indicates the HP-PRRSV 30-aa characteristic discontinuous deletion; and purple indicates some additional a deletions in QYYZ-like PRRSVs. **(B)** Alignment of the deduced amino acid sequence based on the ORF5 gene. The signal peptide and transmembrane (TM) regions are shown in blue and black boxes, respectively. The linear antigenic epitopes and cellular epitopes are indicated in yellow and blue, respectively. The seven amino acids characteristic of QYYZ-like strains are shown in gray.

Nsp2 is the most significant variable region, with remarkable mutations, insertions and deletions, and is recognized as an important target gene for analyzing the genetic variation and molecular epidemiology of PRRSV (34). Partial Nsp2 sequence alignment showed that 37 Nsp2 sequences (from complete genome sequences of sublineage 3.5 PRRSVs) were divided into 5 large patterns (Figure 3A). Compared to Nsp2 of VR2332, the Nsp2 proteins of pattern A strains had a deletion pattern that was identical to that of JXA1 (1 aa +29 aa). Pattern B strains not only have the same 30-amino acid (aa) deletion in Nsp2 as JXA1 (1 aa +29 aa) but also have a 36-aa insertion at position 817-852, which was the same as QYYZ (Figure 3A). Pattern C strains also have the same 36-aa insertion in Nsp2 as QYYZ (Figure 3A). Additionally, Pattern D strains contained the discontinuous 131-aa deletion in Nsp2 identical to that in NADC30 (111 + 1 + 19 aa) (Figure 3A). In addition, 13 of the 37 Chinese lineage 3 PRRSV strains (XJzx12015, HiNZWQ, GXXNF78-1806, SH/CH/2016, GZgy17, JX/CH/2016, GD-KP, FJFS, GXXNF10-1803, GXXNF74-1806, GDXNF229-1811, GDsg, and SDWH27-1710) also have special amino acid deletions in the Nsp2 region, and they all have different deletion patterns (Figure 3A). Interestingly, 12 out of 13 strains with special amino acid deletions showed an amino acid deletion at positions 468-518 (Figure 3A). QYYZ-like PRRSV strains appeared to have more amino acid insertions or deletions in the Nsp2 region than other PRRSV strains. They seem to be more prone to amino acid deficiencies at position 468-518 of Nsp2. The Nsp2 hypervariable region (323-521) not only plays an important regulatory role in maintaining the balance of different viral mRNA species but also regulates PRRSV tropism to primary porcine alveolar macrophages (PAMs) (35). Therefore, these amino acid deletions in the Nsp2 region may alter the cellular tropism of the strains.

GP5 is the major envelope protein of PRRSV and is responsible for the lack of immunological cross-protection among different PRRSV strains due to its hypervariability (36–38). Comparison analyses of the amino acid sequence of QYYZ-like GP5 with those of the other representative strains showed that most strains encode 201 aa, but GDXNF229-1811, GDYDZZZ, and GXTZJ2325-2112 have a 1-aa deletion at residue 33 in VR2332, which is identical to the mutation in SD53-1603 (Figure 3B). Seven unique amino acid substitutions, namely, F^25^ → S^25^, A^26^ → I^26^, H^38^→*Y*^38^, L^39^ → S^39^, T^66^ → C^66^, A^92^ → S^92^, and L^152^ → I^152^, compared with the VR2332 strain. These amino acid mutations were identified only in all QYYZ-like strains but no other representative PRRSV strains (Figure 3B). Interestingly, although the QYYZ-like strains have low homology among them, they still showed consistent molecular features, which may be used as molecular markers to distinguish QYYZ-like strains from other type 2 PRRSV strains in China. GP5 is an envelope protein essential for viral infection, and at least three B-cell and two T-cell epitopes were identified for GP5 (39, 40). Two characteristic mutations of QYYZ-like strains, at positions 38(Y^38^) and 39(S^39^), are in epitope B (37-45 aa), which is a highly conserved peptide sequence that presumably functions as a major target for broadly neutralizing antibodies (37, 41). At the same time, there was one amino acid substitution in T-cell epitope 2 (I^152^), compared to PRRSV strains in other lineages (42). We then analyzed the functional domains in GP5 of QYYZ-like strains, including the signal peptide and transmembrane (TM) domain (43). Two residues (S^25^ and I^26^) resided within the signal peptide (aa 1-31). Moreover, there was a unique amino acid mutation at position 66 in the TM region of the QYYZ-like strains, substituting T^66^ to C^66^. Briefly, we found 7 unique amino acid mutations in the GP5 protein, and some mutation sites were located in cell epitopes, the signal peptide and the TM region. These amino acid substitutions might lead to the failure of receptor recognition and thus result in the failure of vaccines.

Recombination is a pervasive phenomenon among PRRSV isolates, and there are an increasing number of reports about the recombination of QYYZ-like PRRSVs (14, 16, 44). In recent years, many recombinant QYYZ-like PRRSVs have reportedly reemerged with increased pathogenicity (12, 18, 34, 45, 46). To identify possible recombination events of the new QYYZ-like PRRSVs in China, RDP4 and SimPlot software were used to assess possible recombinant events (Table 4 and Supplementary Figure 1). This analysis demonstrated that all nine new QYYZ-like isolates were recombinant viruses. Six recombinant isolates (GXXNF10-1803, GXXNF53-1805, GDXNF60-1805, GXXNF74-1806, GXXNF78-1806, and GDXNF229-1811) emerged from recombination events between HP-PRRSV isolates and QYYZ-like virus; three recombinant isolates (HNLCL15-1903, HNTZJ1714-2011, and GXTZJ2325-2112) were also derived from NADC30-like PRRSV, HP-PRRSV isolates, and QYYZ-like PRRSV (Table 4 and Supplementary Figure 1). These putative recombination events were further supported by statistically incongruent phylogenetic trees (Supplementary Figure 1). According to a similarity plot, these nine strains have an extremely complex recombination pattern (Supplementary Figure 1). Interestingly, the HNLCL15-1903 strain has almost the same recombination pattern as the previous strain SCya18 (MK144543.1) and high homology (BLAST analysis: 97.99%). SCya18 was isolated in Sichuan Province in 2018 (29), while HNLCL15-1903 was isolated in Henan Province in 2019. The two provinces are not adjacent, and under the background of low homology and complex recombination of QYYZ-like strains, the emergence of two strains with high similarity is noteworthy.

**Table 4:** Information on recombination events of QYYZ-like PRRSV isolates detected by RDP4 software.

**Strains**	**Breakpoints**		**Parental sequence**		**Detection methods (*p*-value)**						
	**Beginning**	**Ending**	**Minor**	**Major**	**RDP**	**GENECONV**	**BootScan**	**MaxChi**	**Chimera**	**SiScan**	**3Seq**
GXXNF10-1803	1	3,507	JXA1	QYYZ	2.448 × 10^−36^	-	1.505 × 10^−27^	1.677 × 10^−28^	1.081 × 10^−23^	9.166 × 10^−82^	4.440 × 10^−16^
	7,781	12,294	JXA1	QYYZ	2.352 × 10^−20^	4.191 × 10^−10^	1.829 × 10^−19^	8.522 × 10^−11^	6.504 × 10^−5^	-	4.440 × 10^−16^
HNLCL15-1903	1	1,659	SH/CH/2016	NADC30	5.474 × 10^−47^	1.583 × 10^−16^	6.869 × 10^−38^	1.302 × 10^−37^	1.196 × 10^−17^	-	1.110 × 10^−16^
	10,531	14,132	SH/CH/2016	NADC30	4.798 × 10^−13^	4.699 × 10^−8^	3.515 × 10^−13^	2.702 × 10^−10^	9.689 × 10^−3^	-	1.221 × 10^−15^
GXXNF53-1805	12,685	15,314	QYYZ	JXA1	4.068 × 10^−37^	-	4.114 × 10^−34^	1.467 × 10^−13^	3.866 × 10^−12^	-	3.330 × 10^−15^
GDXNF60-1805	12,541	15,316	QYYZ	JXA1	1.153 × 10^−26^	1.115 × 10^−14^	5.567 × 10^−26^	9.478 × 10^−09^	1.709 × 10^−09^	-	4.440 × 10^−16^
GXXNF74-1806	1	2,339	JXA1	QYYZ	3.589 × 10^−85^	5.042 × 10^−53^	2.133 × 10^−82^	1.171 × 10^−31^	7.599 × 10^−35^	-	4.440 × 10^−16^
	6,924	8,969	JXA1	QYYZ	1.197 × 10^−73^	7.929 × 10^−30^	3.125 × 10^−73^	3.349 × 10^−23^	3.736 × 10^−25^	-	1.110 × 10^−16^
GXXNF78-1806	11,987	12,851	QYYZ	JXA1	2.804 × 10^−68^	1.001 × 10^−35^	1.478 × 10^−67^	2.161 × 10^−19^	1.175 × 10^−21^	-	4.440 × 10^−16^
	13,673	15,266	QYYZ	JXA1	4.474 × 10^−83^	6.225 × 10^−53^	1.542 × 10^−80^	1.379 × 10^−28^	6.139 × 10^−30^	-	1.099 × 10^−14^
GDXNF229-1811	1	2,285	JXA1	QYYZ	5.898 × 10^−65^	1.549 × 10^−52^	3.919 × 10^−70^	8.204 × 10^−25^	7.417 × 10^−21^	-	3.330 × 10^−16^
	6,317	8,812	JXA1	QYYZ	6.152 × 10^−86^	2.128 × 10^−72^	1.950 × 10^−86^	8.256 × 10^−27^	2.368 × 10^−21^	-	3.330 × 10^−16^
HNTZJ1714-2011	488	3,548	NADC30	JXA1	8.808 × 10^−125^	2.849 × 10^−88^	7.364 × 10^−54^	2.100 × 10^−50^	8.792 × 10^−58^	1.554 × 10^−50^	4.440 × 10^−15^
	11,668	15,011	QYYZ	JXA1	1.119 × 10^−3^	4.470 × 10^−19^	1.405 × 10^−12^	4.438 × 10^−8^	9.950 × 10^−6^	8.177 × 10^−40^	-
GXTZJ2325-2112	1	2,004	JXA1	QYYZ	1.870 × 10^−12^	-	1.777 × 10^−13^	1.089 × 10^−9^	1.218 × 10^−14^	2.968 × 10^−11^	
	2,004	5,268	NADC30	QYYZ	2.595 × 10^−95^	1.055 × 10^−67^	3.404 × 10^−92^	7.725 × 10^−30^	8.322 × 10^−38^	1.696 × 10^−51^	1.332 × 10^−15^

To better explore the recombination characteristics of QYYZ-like strains in China (Table 3), we summarized the reported genome-wide recombination of all QYYZ-like strains (ORF5 classified into sublineage 3.5). Interestingly, all 37 QYYZ-like PRRSV strains except for QYYZ (the QYYZ-like original strain) have undergone recombination (Table 3 and Supplementary Figure 2B). The distribution of recombination breakpoints is dispersed and complex, there is no obvious recombination hotspot, and there are relatively many recombination breakpoints located in Nsp12(7), Nsp2(13) and GP2(13) (Supplementary Figure 2B). In a previous study in China, it was found that between 2014 and 2018, the high-frequency interlineage (mainly lineage 1 and lineage 8) recombination regions were located in Nsp9 and GP2 to GP3 (20, 31). Obviously, the recombination hot spots of QYYZ-like strains (sublineage 3.5) are not exactly the same as those of lineage 1 and lineage 8 strains. At the same time, it can be clearly seen that QYYZ-like strains mainly provide fragments of structural protein regions (GP2-N) for recombinant strains (Supplementary Figure 2A), while in the Nsp1 region of the sequence, only GM2 is provided by the QYYZ strain (Table 3). We used the Nsp1 region of QYYZ-like strains to construct a phylogenetic tree for verification (Figure 2D). The majority (33/37) of the QYYZ-like strains were grouped into sublineage 8.7 (JXA1-like), and FJLIUY-2017 and HNTZJ1714-2011 were grouped into sublineage 1.8 (NADC30-like). Only GM2 and QYYZ were classified as sublineage 3.5 (QYYZ-like). To obtain better information about the QYYZ-like strains, we suggest that researchers add a pair of primers to sequence the Nsp1 region of the virus when it is identified as a QYYZ-like strain by ORF5 sequencing.

Now, an overwhelming majority of the PRRSV-2 strains in China can be classified into JXA1-like/CH-1a-like (sublineage 8.7), VR2332-like (sublineage 5.1), NADC30-like/NADC34-like (lineage 1), and QYYZ-like (sublineage 3.5). The CH-1a-like strains first appeared in China and then gradually evolved into JXA1-like (HP-PRRSV) strains (45). The JXA1-like strains are also widespread in Cambodia, Thailand, Vietnam and many other Asian countries (6, 47–49). VR2332-like strains containing the Ingelvac PRRS MLV vaccine sequence are the most widespread, with viruses introduced to more than 10 countries (5). Lineage 1 (NADC30-like and NADC34-like) strains are also globally epidemic and have spread to South America, North America and Asia (7, 33, 50–52). The lineage 3 strains originated in Taiwan. Interestingly, after 20 years of transmission and evolution, these strains are prevalent only in greater China (mainland China, Taiwan, and Hong Kong) (18). Moreover, the QYYZ-like strains (sublineage 3.5) were prevalent only in mainland China. According to the results of this study and previous studies, QYYZ-like strains have been occasionally reported in northern China (19, 26, 28, 53) but are mainly prevalent in southern and central China (12–16, 23–25, 27, 29, 30, 32). Although recombination of PRRSV is very common, many non-recombination strains of JXA1-like (54), VR2332-like (28) and NADC30-like/NADC34-like PRRSV (7, 55) have been reported after long-term evolution in China. All QYYZ-like strains were recombined with other lineages of PRRSV except the prototype strain QYYZ. Thus, we speculated that the non-recombination QYYZ-like strains are no longer circulating and that they played a role as a provider of recombination fragments in the PRRSV epidemic in China.

## Conclusion

In summary, QYYZ-like PRRSV strains did not have a large-scale epidemic status but persisted in central and southern China during 2018–2021. QYYZ-like strains have low homology and extremely complex amino acid insertion and deletion patterns in the Nsp2 region. However, they have seven identical amino acid mutations in the GP5 protein. These strains all underwent complex recombination except the prototype strain QYYZ and mainly provided structural protein fragments (GP2-N) for the recombinant strains. These results will help us to understand the overall genomic characteristics of QYYZ-like PRRSV, which is useful for the prevention and control of this virus.

## Data Availability Statement

The original contributions presented in the study are included in the article/Supplementary Material, further inquiries can be directed to the corresponding author/s.

## Ethics Statement

The animal study was reviewed and approved by Sampling procedures were performed in accordance with the guidelines of the Animal Ethics Committee of the School of Harbin Veterinary Research Institute of the Chinese Academy of Agricultural Sciences. The Animal Ethics Committee Approval Number was SYXK(Hei) 2011022.

## Author Contributions

Conceived and designed the experiments: MS, Y-DT, HZ, and Z-JT. Performed the experiments: HX and LX. Contributed reagents or materials and assisted in some experiments: CLi, JZ, BG, QS, JP, QW, GZ, TA, and XC. Analyzed the data: LX, CLe, and Z-JT. Contributed to the writing of the manuscript: HX, LX, and HZ. All authors contributed to the article and approved the submitted version.

## Funding

This study was supported by the National Parasitic Resources Center (NPRC-2019-194-30), the National Natural Science Foundation of China (Grant no. 32172890 and 32002315), and the China Postdoctoral Fund (Grant no. 2020M680788).

## Conflict of Interest

The authors declare that the research was conducted in the absence of any commercial or financial relationships that could be construed as a potential conflict of interest.

## Publisher's Note

All claims expressed in this article are solely those of the authors and do not necessarily represent those of their affiliated organizations, or those of the publisher, the editors and the reviewers. Any product that may be evaluated in this article, or claim that may be made by its manufacturer, is not guaranteed or endorsed by the publisher.

## Supplementary Material

Supplementary Figure 1 Recombination analysis of GXXNF10-1803, GXXNF53-1805, GDXNF60-1805, GXXNF74-1806, GXXNF78-1806, GDXNF229-1811, HNLCL15-1903, HNTZJ1714-2011, and GXTZJ2325-2112. Phylogenic trees were constructed based on different parent regions. Blue represents HP-PRRSV, yellow represents NADC30-like PRRSV, and red represents QYYZ-like PRRSV.

Supplementary Figure 2 Summary of recombination breakpoints of all reported QYYZ strains in China. **(A)** The number of recombinant fragments provided by QYYZ in each region of 37 recombinant strains. The ORF5 of all 37 recombinant strains was provided by the QYYZ strain, while the Nsp1 region was provided by the QYYZ strain in only two strains. **(B)** Recombination breakpoints identified across the full-length genome. The putative recombinants are listed in the key, with the red triangles matching each corresponding breakpoint. Most breakpoints were localized in Nsp2 and GP2, and the backgrounds of the two regions are highlighted in green.
