# Supplementary figures and images for: Genome-Wide Characterization of QYYZ-Like PRRSV During 2018–2021
Source: Front Vet Sci. 2022 Jun 30;9:945381. doi: 10.3389/fvets.2022.945381 (PMC9280713; doi:10.3389/fvets.2022.945381)

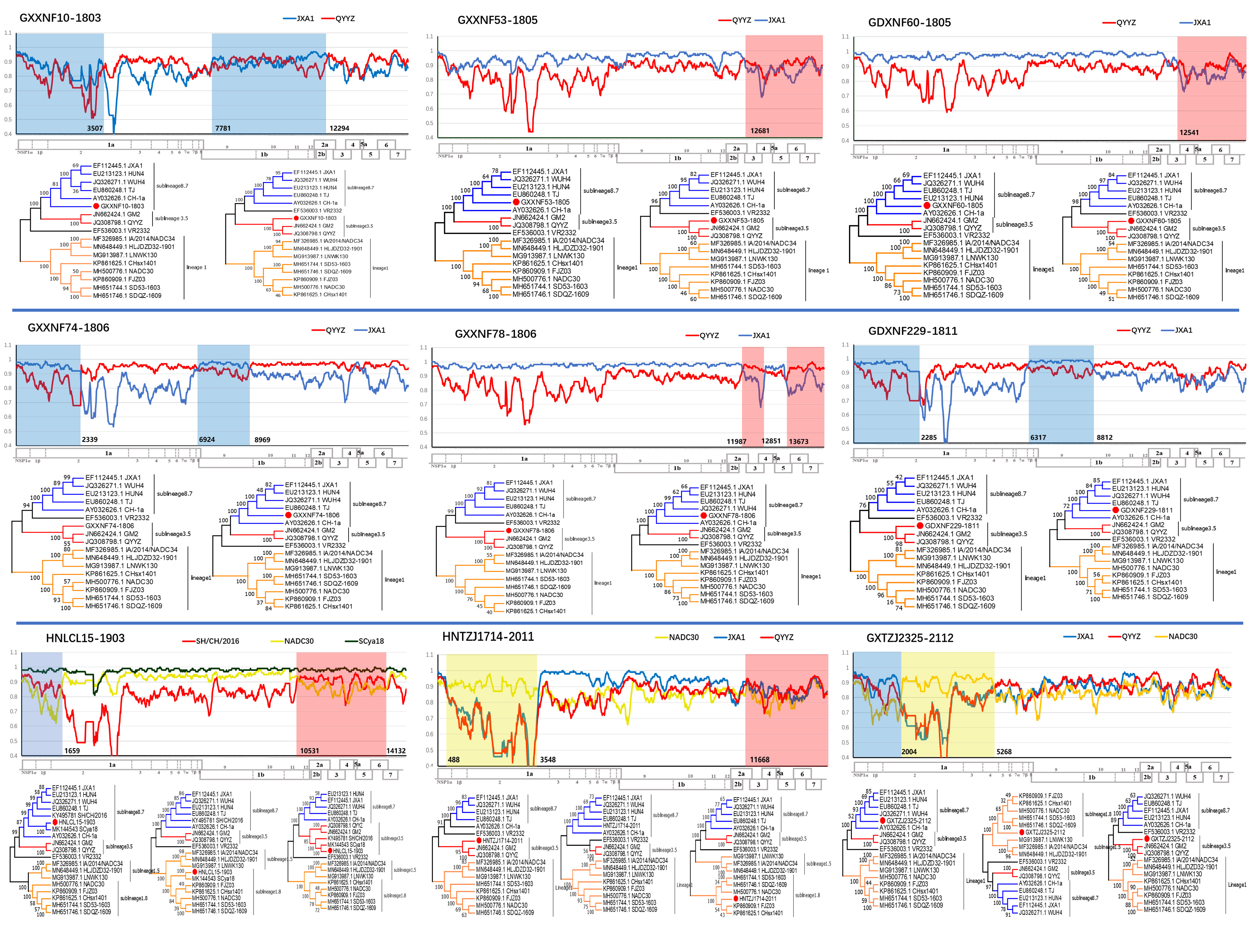

Supplement: Supplementary Figure 1 — Recombination analysis of GXXNF10-1803, GXXNF53-1805, GDXNF60-1805, GXXNF74-1806, GXXNF78-1806, GDXNF229-1811, HNLCL15-1903, HNTZJ1714-2011, and GXTZJ2325-2112. Phylogenic trees were constructed based on different parent regions. Blue represents HP-PRRSV, yellow represents NADC30-like PRRSV, and red represents QYYZ-like PRRSV. [file Image_1.JPG]

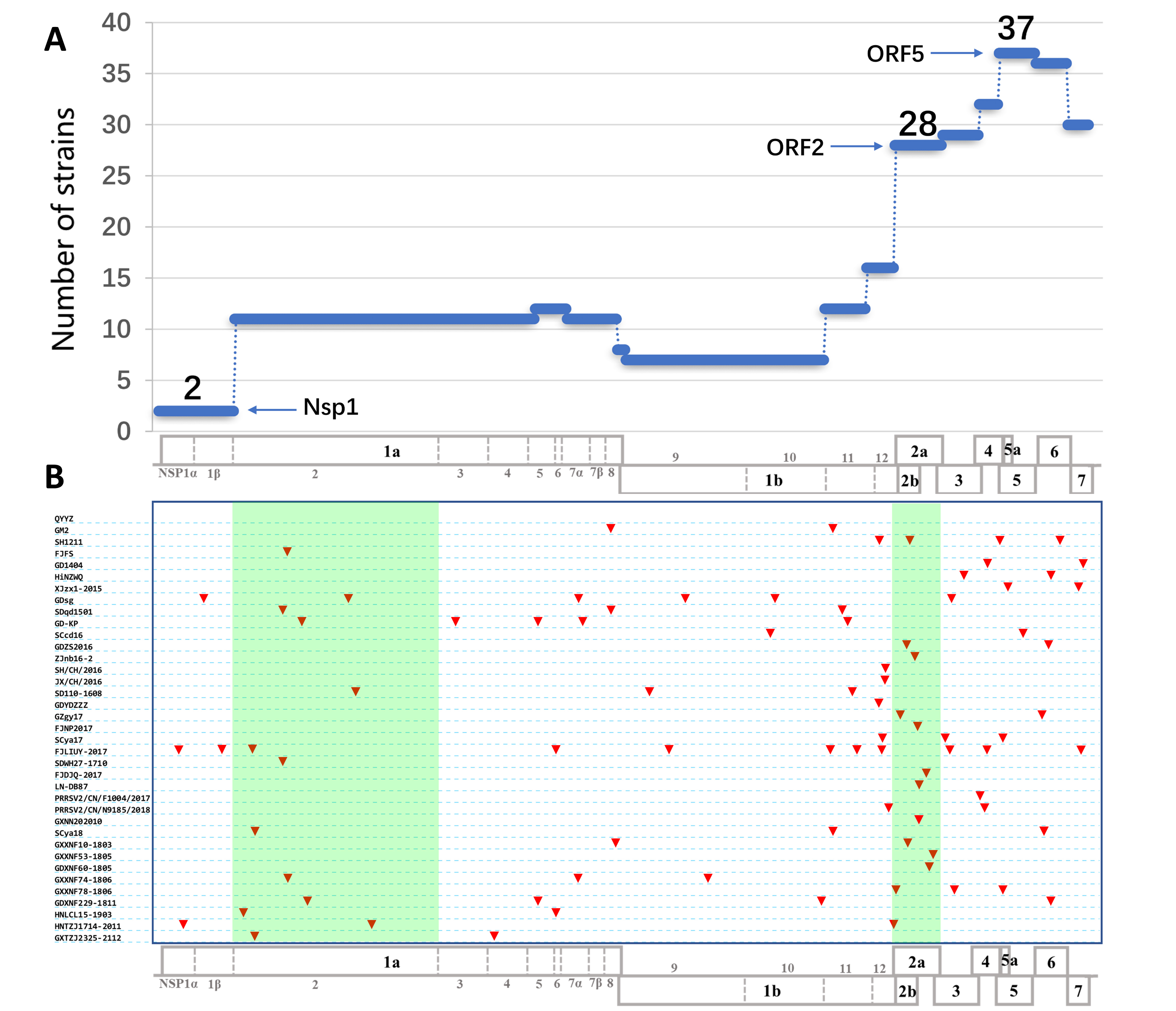

Supplement: Supplementary Figure 2 — Summary of recombination breakpoints of all reported QYYZ strains in China. (A) The number of recombinant fragments provided by QYYZ in each region of 37 recombinant strains. The ORF5 of all 37 recombinant strains was provided by the QYYZ strain, while the Nsp1 region was provided by the QYYZ strain in only two strains. (B) Recombination breakpoints identified across the full-length genome. The putative recombinants are listed in the key, with the red triangles matching each corresponding breakpoint. Most breakpoints were localized in Nsp2 and GP2, and the backgrounds of the two regions are highlighted in green. [file Image_2.JPG]
